# Evaluation of RT-PCR assays for detection of SARS-CoV-2 variants of concern

**DOI:** 10.1038/s41598-023-28275-y

**Published:** 2023-02-09

**Authors:** Sourav Dutta Dip, Shovon Lal Sarkar, Md. Ali Ahasan Setu, Prosanto Kumar Das, Md. Hasan Ali Pramanik, A. S. M. Rubayet Ul Alam, Hassan M. Al-Emran, M. Anwar Hossain, Iqbal Kabir Jahid

**Affiliations:** 1 Department of Microbiology, Jashore University of Science and Technology, Jashore, 7408 Bangladesh; 2 Department of Biomedical Engineering, Jashore University of Science and Technology, Jashore, 7408 Bangladesh; 3 Department of Microbiology, University of Dhaka, Dhaka, 1000 Bangladesh; 4 Genome Center, Jashore University of Science and Technology, Jashore, 7408 Bangladesh

**Keywords:** SARS-CoV-2, Infectious-disease diagnostics, Policy and public health in microbiology, Biological techniques, Microbiology, Molecular biology

## Abstract

Severe Acute Respiratory Syndrome Coronavirus-2 (SARS-CoV-2) pandemic has been considered with great importance on correct screening procedure. The detection efficiency of recent variants of concern were observed by comparing 5 commercial RT-PCR kits and a SYBR-green method developed and validated in our laboratory. The RNA was extracted from nasopharyngeal samples from suspected COVID-19 patients and RT-PCR assay was performed according to the instruction of the respective manufacturers. The specificity and sensitivity of Maccura kit was 81.8% and 82.5%, A*Star kit was 100% and 75.4%, Da An Gene kit was 100% and 68.4%, Sansure kit was 54.5% and 91.2% and TaqPath kit was 100% and 70.2% respectively. Our in house SYBR-Green method showed a consistent detection result with 90.9% specificity and 91.2% sensitivity. We also found that detection kits targeting more genes showed better accuracy which facilitates less false positive results (< 20%). Our study found a significant difference (p < 0.005) in Ct value reported for common target genes shared by the RT-PCR kits in relation with different variants of COVID-19 infection. Recent variants of concerns contain more than 30 mutations in the spike proteins including 2 deletion and a unique insertion mutation by which makes detection of these variants difficult and these facilitates the variants to escape from being detected.

## Introduction

The first reported case of severe acute respiratory syndrome coronavirus-2 (SARS-CoV-2) was in Wuhan, city of Hubei province, China, on 8th December 2019, as a cluster of pneumonia of unknown etiology by Wuhan Municipal Health and Health Commission^1–3^. The virus has spread globally due to its high transmission capability and the World Health Organization (WHO) declared it as a pandemic on 11th March, 2021^4^. The worldwide recorded nearly 633 million confirmed cases and 6.6 million deaths as of March 18, 2022^5^. SARS-CoV-2 is a positive sense single-stranded RNA virus with genome size approximately 29 kb^6^. The genome contains 14 open reading frames encoding structural proteins including Spike (S), Envelope (E), Nucleocapsid (N), Membrane (M) along with eight accessory proteins and nonstructural proteins (NSPs) like RNA-dependent RNA polymerase (RdRp) protein^6,7^. The RT-PCR is the most reliable and considered as the gold standard widely all over the world for the diagnosis of SARS-CoV-2^2,8^. The assays have been developed targeting highly conserved regions of ORF1ab, N, S, E, RNA-Dependent RNA-polymerase (RdRp) gene^9,10^. Most of the commercially available RT-PCR kits are using ORF1ab and N gene as the detection target^11^. Though ORF1ab is the highest specific target for confirming SARS-CoV-2, it is showing less sensitivity than other gene targets in the RT-PCR assay; for which current RT-PCR assays not only have a high false-negative rate but also a low sensitivity rate^10^. As of October, 2022; “FIND diagnostic for all” organization enlisted 139 SARS-CoV-2 RT-PCR kits commercialized in different countries^12^. In this study, we have performed evaluation of five commercially available RT-PCR assays along with an in-house SYBR-Green method of the Genome center, Jashore University of Science and Technology. Among these assays, Maccura (SARS-CoV-2 fluorescent PCR kit) and TaqPath™ COVID-19 CE-IVD RT-PCR Kit uses three genes as a target whereas other kits use two genes except for A*Star Fortitude kit 2.0, which used only one gene as a target. We additionally performed Sanger sequencing of spike receptor-binding region to determine whether the recent Variants of Concern (VOCs) remain undetected by any of the RT-PCR assays.

## Results

### Sensitivity and specificity assessment

The sensitivity and specificity assessment based on 57 positive samples and 33 negative samples is demonstrated in Table 1. All of the kits showed a high specificity except the Sansure kit that showed the lowest of 54.5%. The A*Star kit, Da An Gene kit and TaqPath kit showed the highest specificity of 100% whereas our in-house SYBR-Green method showed the specificity of 91.0% and Maccura kit showed a specificity of 81.8%. Sensitivity was found highest for Sansure and SYBR-green method of 91.2%; the lowest sensitivity was shown by the Da An Gene kit of 68.4%. The Maccura kit showed the sensitivity of 82.5%; A*Star kit showed 75.4% and TaqPath kit showed 70.2% respectively. All of the kits showed a substantial Inter-Rater Agreement (Kappa value) with the gold standard considered, whereas the Sansure kit showed a moderate agreement. The SYBR-Green method showed the highest agreement of 0.81; followed by the A*Star kit showing 0.69; the TaqPath kit showing 0.63; the Maccura kit showing 0.63; the Da An gene kit showing 0.61 and the Sansure kit showing he lowest of 0.49. All of the kits showed a diagnostic accuracy over 80%; the SYBR-Green method showed the highest of 91.1%; the A*Star kit showed the accuracy of 84.0%; the Maccura kit showed 82.2%; the TaqPath kit showed 81.1%; the Da An Gene kit showed 80.0%, whereas the Sansure kit showed the lowest of 77.7%. Sansure kit also showed the highest False Positive Rate of 45.5% whereas the TaqPath kit, the Da An Gene kit and the A*Star kit showed 0%; SYBR-Green method showed a 9.1% false positive result and the Maccura kit showed 18.2%. The RT-PCR kits showed a variety of false negative rate with the highest of 31.6% showed by Da An Gene. The lowest False negative rate was showed by Sansure kit and SYBR-Green method (8.8%) followed by the Maccura kit (17.5%); the A*Star kit (24.6%) and the Taqpath kit (29.8%). The Positive Predictive Value (PPV) and Negative Predictive Value (NPV) for the Maccura kit was 88.7% and 73.0%; for A*Star kit it was 100% and 70.2%; for the Da An Gene kit 100% and 57.0%; for Sansure kit it was 77.6% and 78.3%; for SYBR-Green method it was 94.5% and 85.71%; for the TaqPath kit it was 100% and 66.0% respectively (Table 1).

**Table 1:** Total run time required for completing a full RT-PCR amplification.

RT-PCR kit name	Time for RNA extraction (per sample)	Total run time required
Maccura (SARS CoV-2 fluorescent PCR kit)	Approximately 40 min	Approximately 1 h 20 min
A*Star Fortitude kit 2.0	Approximately 27 min 30 s	Approximately 1 h 15 min
Da An Gene Co. Ltd. of SunYat-Sen University	Approximately 5 min	Approximately 1 h 33 min 24 s
Novel Coronavirus (2019-nCoV) Nucleic Acid Diagnostic Test (PCR-Fluorescent Probing) from Sansure Biotech	Approximately 17 min	Approximately 1 h 28 min 26 s
SYBR-Green Based detection primers designed in our Laboratory	Approximately 17 min	1 h 31 min 34 s
TaqPath™ COVID-19 CE-IVD RT-PCR Kit	Approximately 40 min	1 h 21 min 37 s

### Comparative assessment of detection result

**Figure 1:**
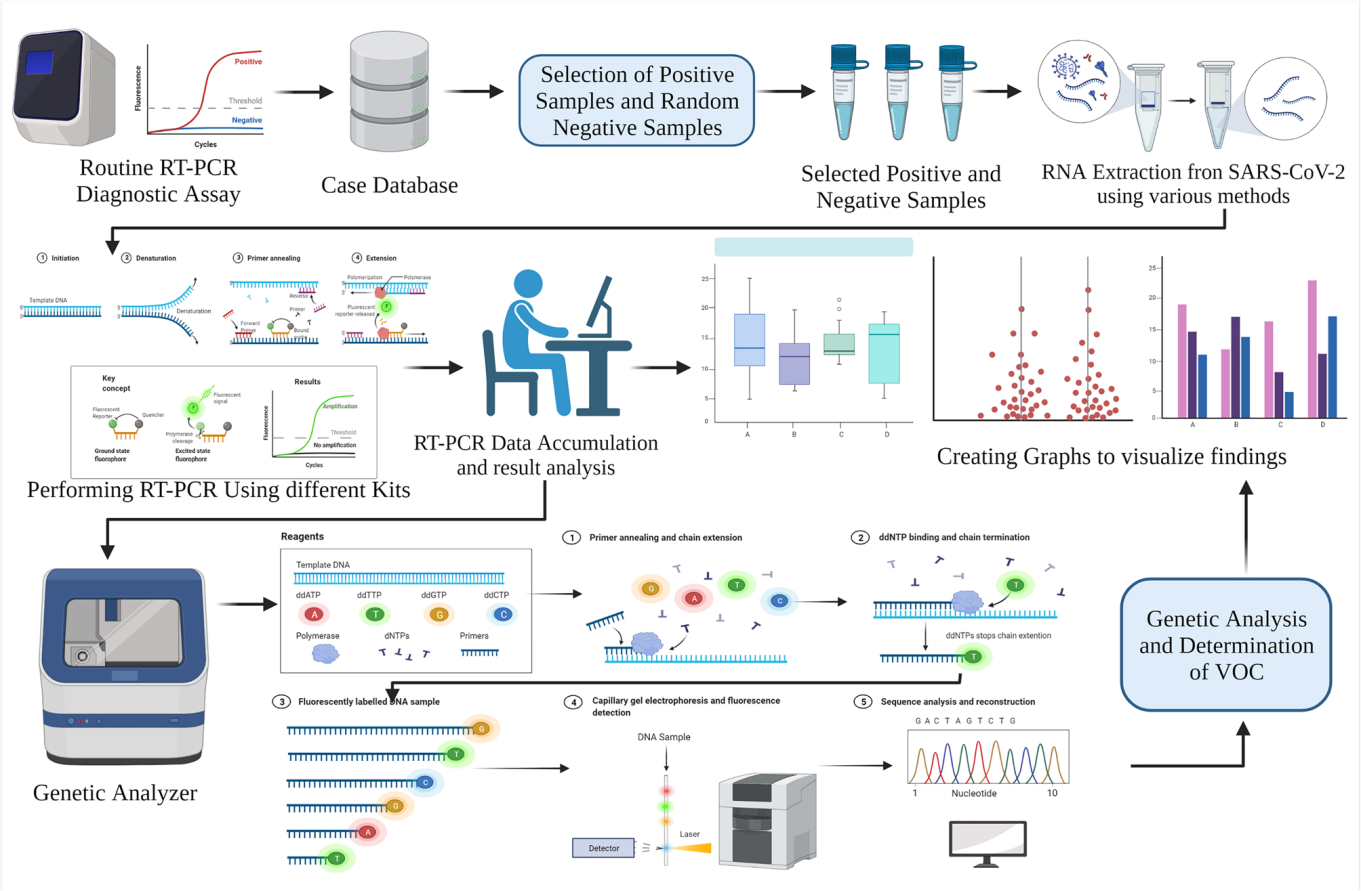
Schematic Workflow of the Evaluation of RT-PCR Assays in detection of SARS-CoV-2 Variant of Concerns. The Upper Portion depicts the sample selection and extraction procedure and the lower portion shows the overall workflow from performing RT-PCR to Data analysis. RT-PCR, Real-Time Polymerase Chain Reaction; VOC, Variant of concern; SARS-CoV-2, Severe Acute Respiratory Syndrome Coronavirus-2.

Samples were considered positive if the Ct value was below the cut-off value as the manufacturers recommended. All 6 considered RT-PCR kits detected all of the Alpha (B.1.1.7) variant showing a 100% detection rate for the variant. whereas for variant Beta (B.1.351) the Sansure kit, the A*Star kit and SYBR-Green method showed 13.3% false negative result; the Maccura kit showed 20% false negative result; the TaqPath kit and the Da An Gene kit both showed 46.7% false negative result. For Wild type variant the Sansure kit, Maccura kit and SYBR-Green method showed 0% false negative result for respective variant; whereas Da An Gene kit, TaqPath kit and A*Star kit showed 16.7% false negative results. Respectively, for Delta (B.1.617.2) variant Sansure kit and SYBR-Green method showed 10% false negative result, the Maccura kit showed 23.3%, the TaqPath kit showed 30%, Da An Gene showed 33.3% and A*Star kit showed 36.7% false negative results. For TaqPath kit it was found S-Gene Target failure for 83.3% Alpha (B.1.1.7) variant samples; 46.7% Beta (B.1.351) variant samples; 33.3% wild type samples but 0% for Delta (B.1.617.2) variant samples. Figure 1 shows the summary of the detection results by the RT-PCR kits as by the sample. In SYBR-Green method the results were interpreted according to the melting curve analysis; cumulative results and peaks were shown in supplementary figure. Peaks shown in supplementary figure (b) shows if the samples are positive. The peaks are for the melting point of detecting gene that confirms positive samples; and supplementary figure (c) depicts the melting point for the housekeeping gene (Beta-Actin) considered for the SYBR-Green method. A single peak for the housekeeping gene indicates that the sample is negative.

### Sanger sequencing

The amplified spike target products were passed through amplicon validation and then identified and confirmed SARS-CoV-2 variants by determining the mutations in specified locations in the spike protein by matching with SARS-CoV-2 spike in both BLAST (> 99%) and MEGA7 based sequence alignment. Of the total 57 samples, 6 samples were found wild type, 30 samples were found with L452R and T478K mutation which confirmed Delta (B.1.617.2) variant. 15 samples were found with K417N, E484K, N501Y mutation confirming Beta (B.1.351) variant and 6 samples were found with E484K, N501Y, D614G mutations confirming Alpha (B.1.1.7) variant.

### Qualitative analysis of Ct value

**Figure 2:**
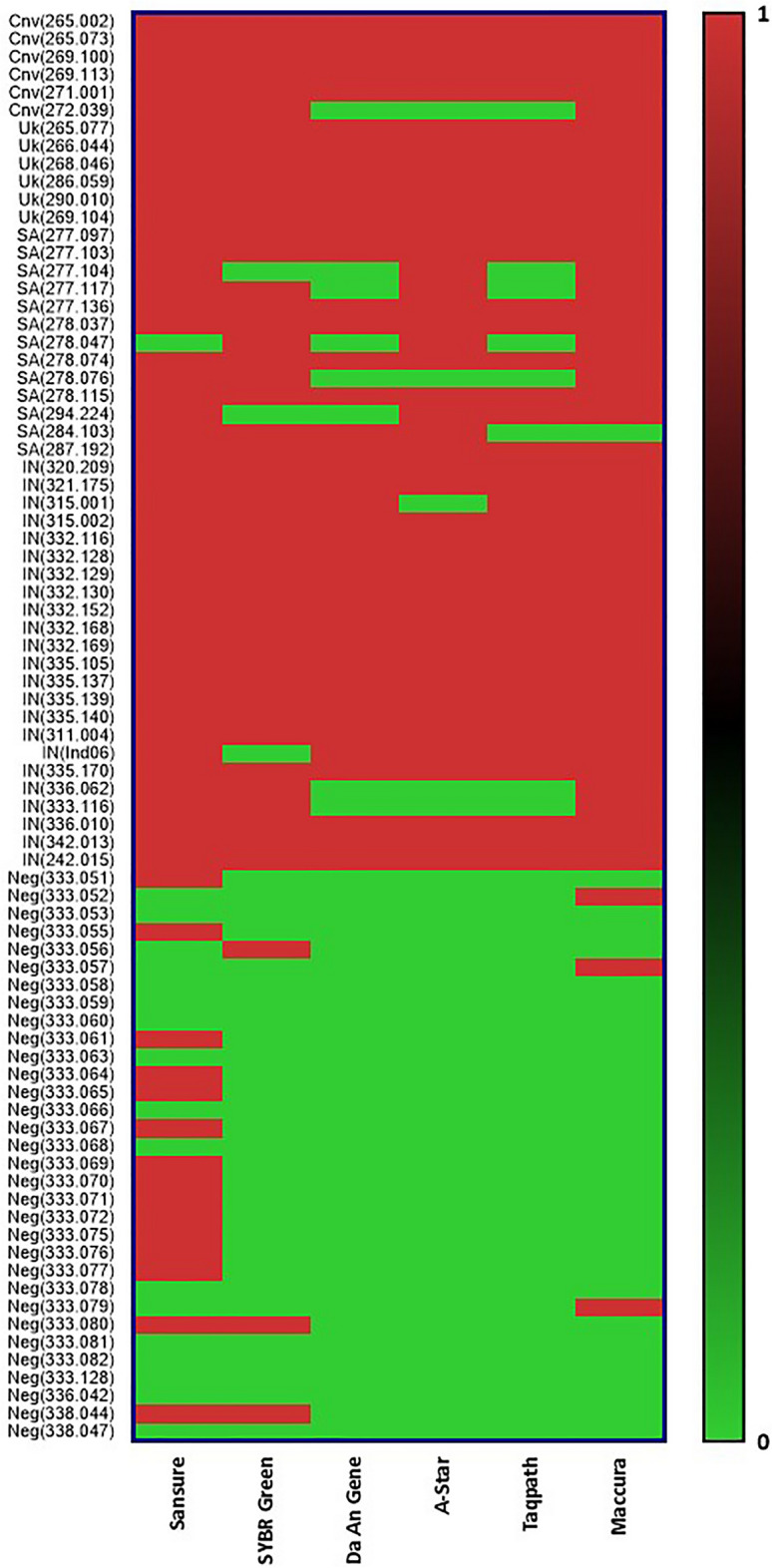
Visualization of screening positive and negative samples is associated with SARS-C0V-2 variants of concern. Screening positive samples represented as “1” & negative samples represented as “0”. SARS-CoV-2 variant shown as Cnv = Conventional/Wild Type, UK = UK variant (ALPHA, B.1.1.7), SA = South Africa Variant (BETA, B.1.351), IN = Indian Variant (DELTA, B.1.617.2) and Neg = Negative. Further confirmed by Sanger sequencing.

**Figure 3:**
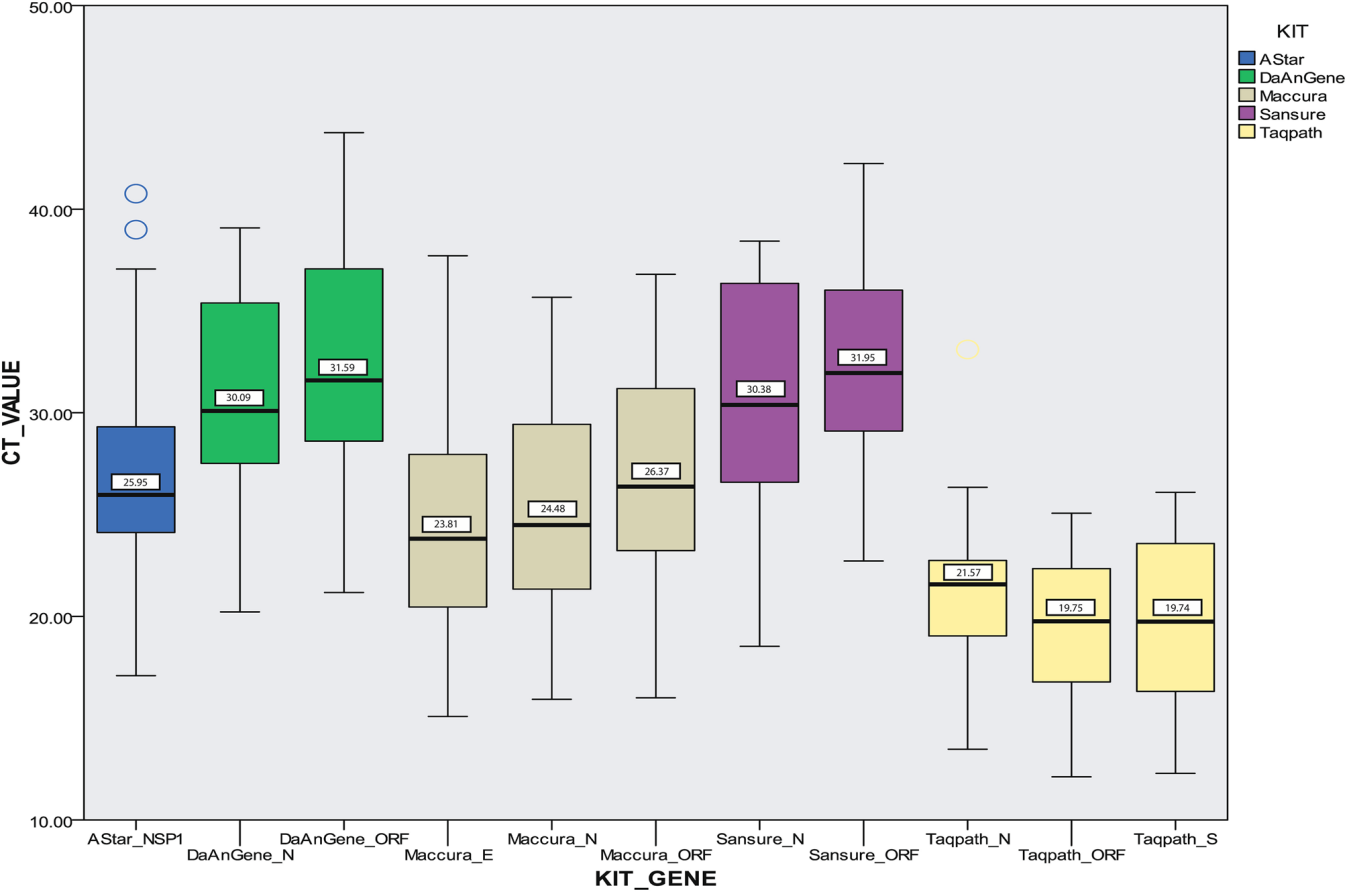
Distribution of Ct value according to their target genes reported by the five commercial kits. The whiskers above boxplot represent the upper limit of the 95% confidence interval (CI) and the whisker below represents the lower limit of 95% confidence interval. The numbers mentioned in the chart represent the median Ct value reported by the kits for their target gene. Sansure and Da An Gene kit tend to give higher Ct value for detection whereas TaqPath Kit tends to show a lower Ct value.

Qualitative analysis of the Ct value reported by the kits for their target genes showed a difference in them. TaqPath kit, Maccura kit, Sansure kit and Da An Gene kit had two common target genes (N, ORF1ab), whereas A*Star kit targeted NSP-1 gene only and TaqPath kit targeted S gene and Maccura kit targeted E gene unlike others. The results show that kit Sansure (Ct = 30.4 for N gene; Ct = 32.0 for ORF gene) and Da An Gene (Ct = 30.1 for N gene and 31.6 for ORF gene) tends to show the highest median Ct value for their target genes followed by A*Star kit, Maccura kit and TaqPath kit. Figure 2 shows the distribution and median values of the Ct value reported and Fig. 3a,b shows the frequency of Ct value reported by the kits.

### Ct value reported by variants of concern

**Figure 4:**
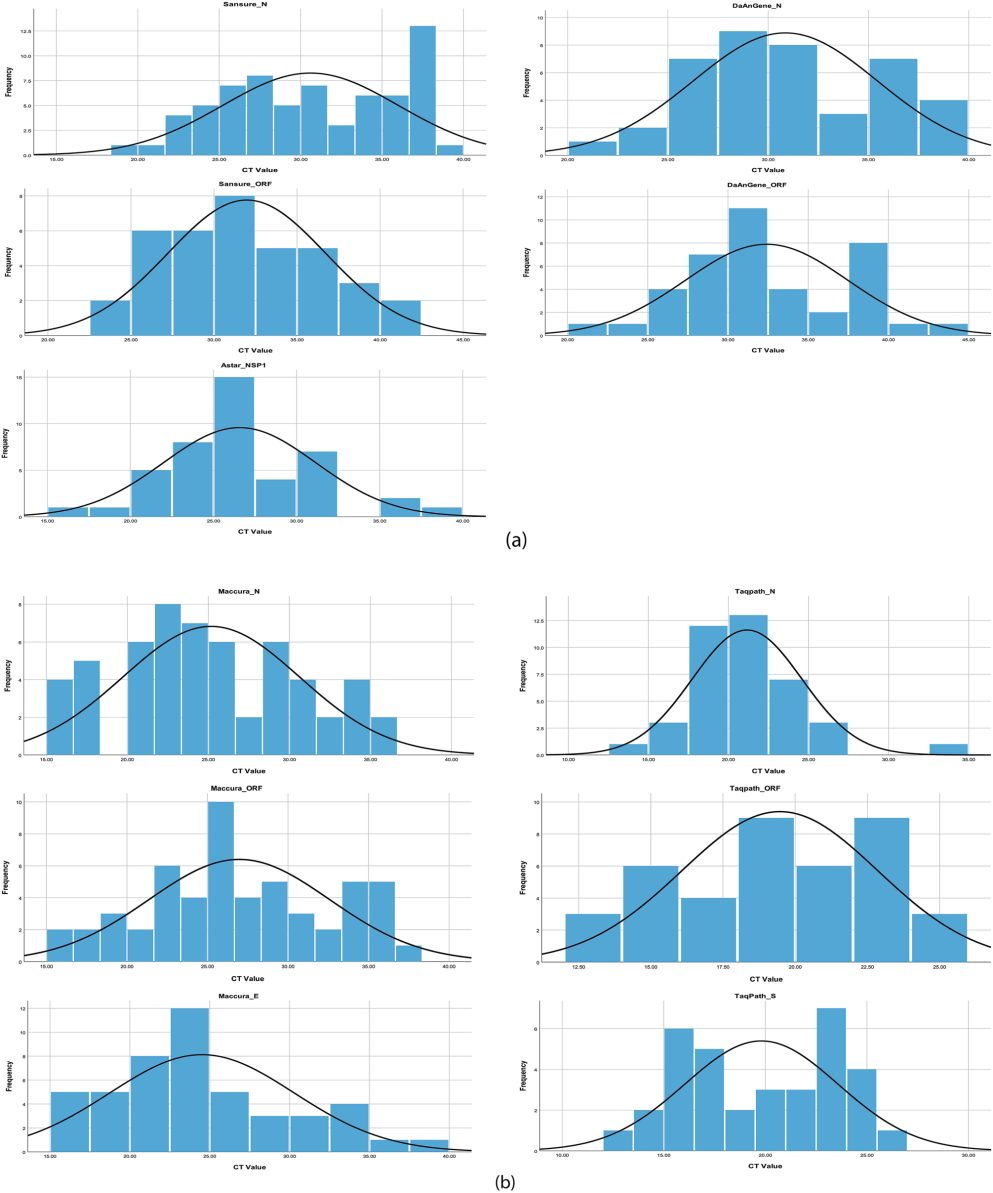
Frequency of Ct value reported by the Kits for their target genes with normal curves. (**a**) Frequency of Ct value for target genes of Sansure, Da An Gene and A*Star; (**b**) Frequency of Ct value for target genes of Maccura and TaqPath.

The summary of Ct value for N gene and ORF1ab gene reported for VOC in different kits were demonstrated in Fig. 4a,b. ANOVA test showed that the Ct value for both N gene (F = 5.359, p < 0.001) and ORF1ab gene (F = 3.198, p < 0.001) were significantly different and specifically variant B.1.351 (Beta, SA variant) and B.1.617.2 (Delta, IND variant) showed higher or undetermined Ct value for both N and ORF1ab in different kits, followed by variant B.1.1.7 (Alpha, UK variant) in comparison with wild type SARS-CoV-2.

## Discussion

SARS-CoV-2 variants are now a major concern in terms of infection rate, travel embargo, and other restrictions. Alpha (B.1.1.7) variant, the first of highly publicized variants, distinguished as VOC in December, 2020^5^. At that time, more than 70% of infections in the UK (98%), USA (70%), Germany (95%), Denmark (99%), Japan (92%), Sweden (97%), Netherland (95%), Italy (91%), Switzerland, Poland (99%), Israel (99%), Qatar (80%), Australia (70%) etc. were reported as Alpha variant^13–15^. Then, this variant faded away in late 2021 due to emergence of a more aggressive Delta (B.1.617.2) variant^16,17^. Another VOC, Beta (B.1.351) variant also emerged during the period of Alpha dominance. But the variant spread less than the Alpha variant. The variant was dominant in mainly South Africa (99%), Bangladesh (85%), Qatar (79%)^13,14^. There were reported infections in other countries as well but not as these countries mentioned. The Delta variant was identified as VOC in May, 2021^5^. During that period more than 70% of the infections were reported as Delta variants in India, UK, USA, Bangladesh, Singapore etc. By July, 2021 more than 90% of infections were to be reported as Delta variant which remained dominant till early 2022 which faded away by the rise of Omicron (B.1.1.529) variant^13,14^.

A accurate diagnosis of the new variants became a challenge since a large number of mutations were increasingly being spotted in the VOCs^18,19^. Unknown primers (patented or trademarked by companies) added more puzzle to the problem since it was often difficult to find out why particular diagnostic kits were not able to produce accurate results^20,21^. Although we observed that missing a gene specially Spike, N or E can be compensated by the presence of another or more gene specific amplicons, and it was expected to check more than one gene specific products during the assay especially for low viral load samples. Some studies suggested that Omicron variant contains more than 30 mutations in its spike protein of which 2 significant deletion mutation (del69-70 and K417N) and a unique insertion mutation (ins214EPE) which hindered the detection procedure^16,22,23^.

In this study, we demonstrated that our in-house assay diagnosed SARS-CoV-2 viruses with better results in terms of both sensitivity and specificity (91.2% each) independent of different variants. This method showed inter-test agreement with the criteria considered for the reference standard than other RT-PCR kits (Table 1). Other kits showed a suboptimal agreement with the reference (Kappa < 0.8) except the Sansure kit which showed a moderate agreement with the reference standard (Kappa < 0.6). Again, according to the results obtained in our study, all of the kits showed a decent diagnostic accuracy over 80% except the Sansure kit which showed 77.77%. The SYBR Green method also showed a decent false positive and false negative rate but the Sansure kit showed the highest false positive rate and Da An Gene showed the highest false negative rate (Table 1). Overall, it can be stated from our obtained result that N and ORF targeting kits tend to show higher amount of false positive result whereas kits depending on higher number of target genes show less false positive results (Table 1).

There was a difference among the Ct value reported by the RT-PCR kits for same sample with same target gene but when compared it showed Sansure kit and Da An Gene kit tend to give higher Ct value for detection whereas TaqPath kit and Maccura Kit tends to show lower and similar reported Ct value for any target gene independent of VOCs that concludes with better detection capability (Fig. 2). SYBR Green Method also showed good consistency in detecting SARS-CoV-2 independent of VOCs as per TaqPath kit and Maccura kit showed. When the Ct value reported by the kits were compared among VOCs, it showed that variants of pangolin lineage B.1.351 (Beta, SA variant) provided higher Ct value or remain undetermined, closely followed by B.1.617.2 (Delta, IND variant) and then B.1.1.7 (Alpha, UK variant) in relation with the wild type of SARS-CoV-2 (Fig. 4).

In our study, B.1.351 (Beta, SA variant) escaped the diagnosis the most, followed by B.1.617.2 (Delta, IND variant), given for any kit (Table 2). We have observed that samples containing B.1.1.7 (Alpha, UK variant) showed the highest (n = 5 out of 6) tendency to remain undetected for S-gene target followed by B.1.351 (Beta, SA variant) (n = 7 out of 15). S-Gene Target Failure (SGTF) is a result which can be possible if the primer set misses the S-gene target from the viral genome and amplification doesn’t occur due to PCR. S-Gene Target Failure (SGTF) is considered as the marker for detection of B.1.1.7 (Alpha, UK variant) and recent Omicron cases are also seen to show S-Gene Target Failure (SGTF) among SARS-CoV-2 isolates. SYBR Green procedure showed better result in the detection which might be due to better attachment of the primer set and the target region of the viral genome. Here we suggest for detecting with higher target genes including both S and E gene along with N and ORF because only S as target gene can lead to S-Gene Target Failure (SGTF) which can be a possible mechanism for B.1.1.7 (Alpha, UK variant) and B.1.351 (Beta, SA variant) to avoid detection according to our study performed as most samples containing these variants showed higher Ct value or remained undetected.

**Table 2:** SARS-CoV-2 Variant-wise detection result and percentages.

RT-PCR Kit name	VOC→	ALPHA, B.1.1.7 (UK)		BETA, B.1.351 (SA)		DELTA, B.1.617.2 (IND)		WILD	
	Total Number→	6		15		30		6	
	Result	Number	Percentage	Number	Percentage	Number	Percentage	Number	Percentage
Novel Coronavirus (2019-nCoV) Nucleic Acid Diagnostic Test (PCR-Fluorescent Probing) from Sansure Biotech.	Positive	6	100	13	86.67	27	90	6	100
	Negative	0	0	2	13.33	3	10	0	0
Da An Gene Co. Ltd. Of SunYat-Sen University	Positive	6	100	8	53.33	20	66.67	5	83.33
	Negative	0	0	7	46.67	10	33.33	1	16.67
Maccura (SARS-CoV-2 Fluorescent PCR Kit)	Positive	6	100	12	80	23	76.67	6	100
	Negative	0	0	3	20	7	23.33	0	0
TaqPath^™^ COVID-19 CE-IVD RT-PCR Kit	Positive	6	100	8	53.33	21	70	5	83.33
	Negative	0	0	7	46.67	9	30	1	16.67
A^*^STAR FORTITUDE KIT 2.0r	Positive	6	100	13	86.67	19	63.33	5	83.33
	Negative	0	0	2	13.33	11	36.67	1	16.67
SYBR-Green method developed in our Laboratory	Positive	6	100	13	86.67	27	90	6	100
	Negative	0	0	2	13.33	3	10	0	0

There were some results that demonstrated negative in Sanger sequencing but positive in some RT-PCR kits (SYBR-n = 2, Sansure-n = 15, Maccura-n = 2). Sanger sequencing required higher viral load remaining in the sample than RT-PCR detection. There were also differences in the sample volume and elution volume in the extraction procedures recommended and also there were differences in the efficiency of nucleic acid extraction kits, which can also facilitate the differences in the results reported by the RT-PCR kits (Table 3).

**Table 3:** The basic information and technique index of selected RT-PCR Kits;

RT-PCR kit name	Manufacturer and country	Extraction methods recommended by the manufacturers	Samples needed as per extraction method	Elution volume of RNA	Final reaction volume for the PCR amplification	Dye and target genes	Thermal cycler equipment used
Maccura (SARS CoV-2 fluorescent PCR kit) (Lot 0420151 Ref.-EGN7103109)	Maccura Biotechnology (USA)	MagBind RNA extraction kit (Lot-0320061, Ref-A42359)	200 μL	35-50μL	40 µL (20 µL sample + 20 µL PCR mix)	FAM(ORF), ROX(E), Cy5(N), HEX/VIC(IC)	Analytik Jena qTOWER^3^G
A*Star Fortitude kit 2.0. (Lot-200301; Ref-NFIH001)	The Agency for Science, Technology and Research (A*STAR), and Tan Tock Seng Hospital (TTSH), Singapore	Qiagen viral RNA mini kit (Lot-56604826, Ref-52906) Purelink Viral RNA/DNA Mini Kit (Lot-2065973, Ref-12280-050) Analytik Jena Instant Virus RNA Kit (Lot-015-17, Ref-845-KS-4250050)	140 μL	35 μL	25 µL (2.5 µL sample + 22.5 µL PCR Mix)	FAM (NSP-1), HEX (IC)	Analytik Jena qTOWER^3^G
Da An Gene Co. Ltd. of SunYat-Sen University (Lot-2020012)	Da An Gene Co., Ltd. of Sun Yat-sen University, China	Da An Gene RNA/DNA purification Kit (Preservation) (Lot-2020013, Ref-20200701)	100 μL (directly mixed into the preservation solution as recommended)	Full solution can be used as extracted RNA sample	25 µL (5 µL sample + 20 µL PCR Mix)	FAM(N), VIC (ORF1ab), Cy5 (Internal Control)	Analytik Jena qTOWER^3^G
Novel Coronavirus (2019-nCoV) Nucleic Acid Diagnostic Test (PCR-Fluorescent Probing) from Sansure Biotech. (Lot-2021117, Ref-S3102E)	Sansure Biotech, China	QuickExtract DNA Extraction Solution 1.0 (Cat No.-QE09050, Lot-21460)	20 μL	Full Solution can be used as extracted RNA sample	25μL (10 μL sample + 15 μL PCR Mix)	FAM (ORF), ROX (N), Cy5 (IC)	Analytik Jena qTOWER^3^G
SYBR-Green method developed in our Laboratory. (Lot-10076186, Ref-E3005L)	Jashore University of Science and Technology,Bangladesh	QuickExtract DNA Extraction Solution 1.0 (Cat No.-QE09050, Lot-21460)	20 μL	Full Solution can be used as extracted RNA sample	20μL (7 μL sample + 13 μL PCR Mix)	SYBR (N, E, Beta Actin as internal control), Detected by Melting Curve Analysis	Applied Biosystems QuantStudio 3 Real-Time PCR System
TaqPath™ COVID-19 CE-IVD RT-PCR Kit. (Lot-2005061, Ref-A48102)	Thermo Fisher Scientific, USA	MagMAX viral/pathogen nucleic acid isolation kit (Lot-00936113, Ref-A42359) Machine used: Kingfisher duo prime for automated RNA extraction	140 μL	40 μL	25μL (10 μL sample + 15 μL PCR Mix)	FAM (ORF), ABY(S), VIC(N), JUN (MS2) [specified for Quantstudio 3 RT-PCR system]	Applied Biosystems QuantStudio 3 Real-Time PCR System

*RT-PCR* real-time polymerase chain reaction.

Our study had a limitation of low number of Alpha and Beta variant of SARS-CoV-2 tested with the selected kits. At the time of our study performed Alpha and Beta variant of SARS-CoV-2 were circulating less and Delta variant was circulating dominantly.

The study demonstrated the diagnostic efficiency and performance of the compared RT-PCR kits regarding its operational aspects. The study also highlighted the inter-test agreement among the detection assays irrespective of target genes or viral load in the sample. Genomic analysis of recent circulating variants showed several mutations in the spike proteins which enabled them not to be detected easily^16,22,23^. For this reason, RT-PCR methods were being prone to giving false-negative results that was very crucial in the early stages infection and screening. Several countries are in need of a more rapid test^24^ and the efficiency of RT-PCR method in diagnosis is being questioned. Because of giving false results and indicating to insufficient, some studies are suggesting other methods (i.e. RT-LAMP) to be more effective than RT-PCR though RT-PCR is considered as the gold standard of COVID-19 detection^24^. But as this procedure is new and comprehensive studies are needed, relying solely on this method may slow down the diagnosis process where RT-PCR is a proven procedure. Our study suggests that RT-PCR assay kit manufacturers needs to put emphasis on developing new and more efficient primer sets rather than depending on previous primer sets to detect these new and unique mutations. Newly and better designed primer sets for regularly considered target genes along with several other genes as target for detection, will facilitate the amplification for better diagnostic accuracy. Thus, mitigating the occurrence of false results specifically in case of VOCs and for better detection accuracy.

## Materials and methods

**Figure 5:**
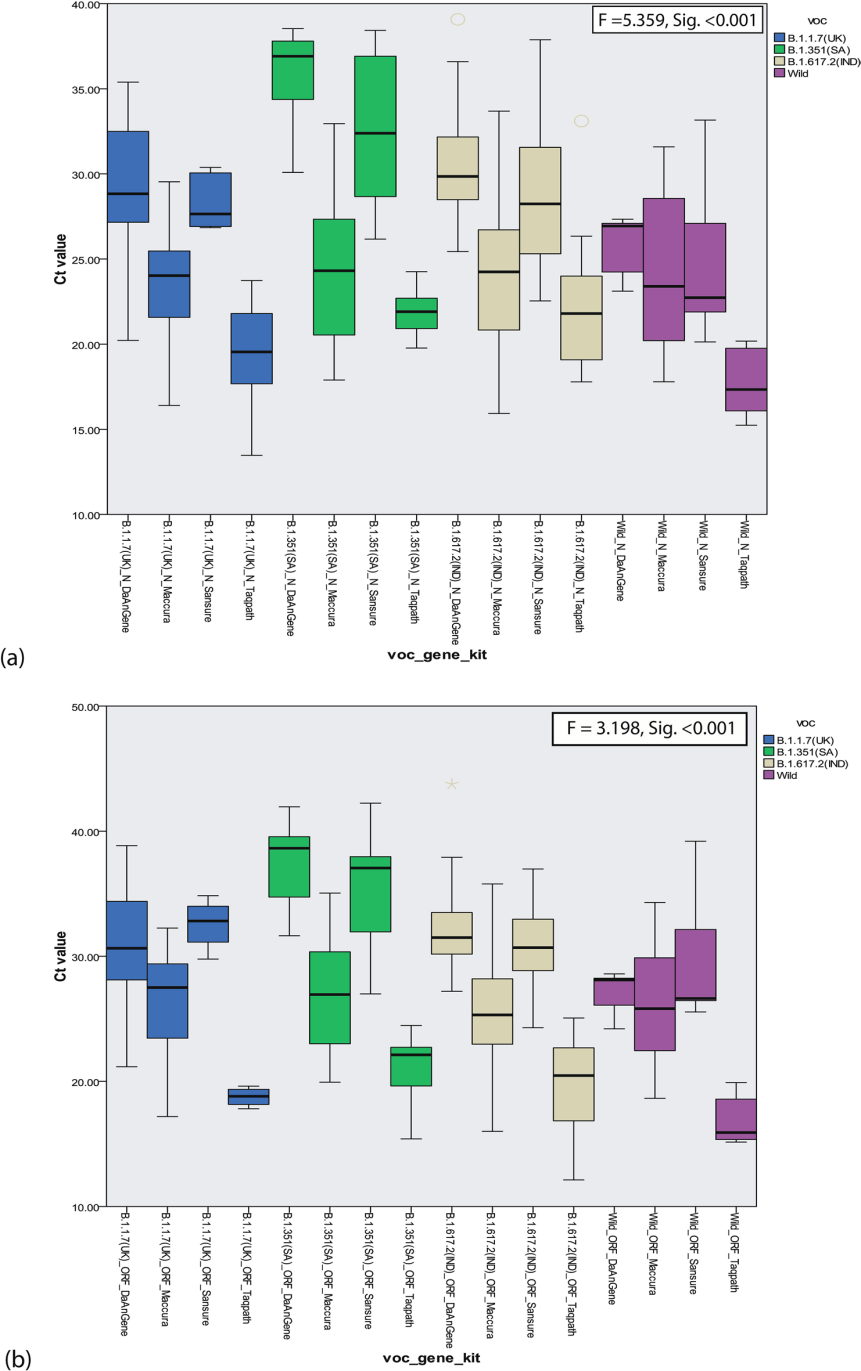
Boxplot showing Ct value reported by variant of concern for target genes of different kits. (**a**) Boxplot of Ct value reported for N-Gene by different Variants of Concern (VOC) in different RT-PCR kits. (**b**) Boxplot of Ct value reported for ORF-Gene by different Variants of Concern (VOCs) in different RT-PCR kits.

The whole study design is demonstrated graphically in Fig. 5. The figure is prepared using a web-based application named BioRender (Agreement Number: EK23H85SRG).

### Sample selection

A Total of 4800 samples were screened from the interval of March, 2021 to June, 2021 at the Genome Center of Jashore University of Science and Technology as a part of national surveillance of COVID-19^25^. Using Sample Size Calculator: Understanding Sample Sizes with a 10% margin of error and 90% confidence interval (CI) level for the samples and finally a total of 90 nasopharyngeal swab samples (Positive and Negative Combined) were selected for the study. All samples were tested from the left-over samples of routine diagnostic services provided for routine COVID-19 surveillance at the Genome Center, Jashore University of Science and Technology (JUST) authorized by the Directorate General of Health Services (DGHS), Bangladesh^26^.

### Selection of RT-PCR kits

Five commercially available COVID-19 RT-PCR kits from the following manufacturers were selected considering the FDA approval and preferences of PCR platforms. In addition to that, our laboratory adopted a validated RT-PCR detection method using SYBR-Green technology. The selected RT-PCR Kits were: (1) Maccura (SARS-CoV-2 Fluorescent PCR Kit), (2) A*STAR FORTITUDE KIT 2.0, (3) Da An Gene Co. Ltd. Of SunYat-Sen University, (4) Novel Coronavirus (2019-nCoV) Nucleic Acid Diagnostic Test (PCR-Fluorescent Probing) from Sansure Biotech, (5) TaqPath™ COVID-19 CE-IVD RT-PCR Kit (Thermofisher) (6) SYBR-Green method developed and validated in Genome Center of Jashore University of Science and Technology^27^. The specification and technical information of all RT-PCR assays were enlisted in Table 3.

### RNA extraction and RT-PCR conditions recommendations by the manufacturers

#### RNA extraction procedures

Nucleic acids were extracted according to the recommendations from the RT-PCR kit manufacturers. For the RT-PCR kits from Sansure Biotech and SYBR-Green method, viral RNA was extracted by QuickExtract™ DNA Extraction Solution 1.0 from Lucigen from which approximately 24 µL of extracted RNA solution was obtained. For A*STAR FORTITUDE KIT 2.0, as purified RNA was recommended, QIamp Viral RNA Mini kit, Purelink Viral RNA/DNA Mini Kit and Analytic Jena Instant Virus RNA Kit was used, that produced approximately 35 µL of RNA eluent. For Maccura RT-PCR kit, recommended Mag-Bind RNA Extraction Kit was used which also produced approximately 35 µL of RNA eluent. For Taqpath RT-PCR Assay, MagMAX viral/Pathogen Nucleic Acid Isolation Kit was used that produced 40 µL of RNA eluent from Automated RNA extractor (Kingfisher Duo Prime). For Da An Gene Co. Ltd. At SunYat-Sen University, The Nucleic Acid extraction method specialized for this specific kit was followed. The specimen was mixed with the lysis buffer provided with the kit, which was later used as the sample for further procedures.

### Thermal cycling condition and standards for result interpretation

Extracted nucleic acids were used to perform RT-PCR and all parameters including, the thermal cycling conditions, reaction volume, detecting channels and dyes etc. were set according to the standards specified by the manufacturers which are shown in Table 4 and Supplementary Table 1. The result was interpreted according to the standards mentioned in the RT-PCR kit manuals summarized in Table 2.

**Table 4:** Sensitivity and specificity test result for different RT-PCR kits.

RT-PCR kit name	Maccura (SARS-CoV-2 fluorescent PCR kit)	A*STAR FORTITUDE KIT 2.0	Da An Gene Co. Ltd. of SunYat-Sen University	Novel coronavirus (2019-nCoV) nucleic acid diagnostic test (PCR-fluorescent probing) from Sansure Biotech.	SYBR-green method developed in our laboratory	TaqPath™ COVID-19 CE-IVD RT-PCR kit
Specificity (%)	81.80	100	100	54.50	90.90	100
Sensitivity (%)	82.50	75.40	68.40	91.20	91.20	70.20
Kappa value	0.627	0.693	0.614	0.489	0.811	0.633
Diagnostic accuracy (%)	82.2	84.0	80.0	77.77	91.1	81.1
Positive predictive value (PPV) %	88.67	100	100	77.62	94.54	100
Negative predictive value (NPV) %	72.97	70.21	56.9	78.26	85.71	66.0
False positive rate (%)	18.2	0	0	45.5	9.1	0
False negative rate (%)	17.5	24.6	31.6	8.8	8.8	29.8
Pearson chi-square value	35.664^a^	47.671^b^	39.845^c^	23.017^d^	59.331^e^	41.684^f^

For Pearson’s chi-square test the P value (Asymptotic Sig. value-2 sided) was < 0.001.

^a^0 cells (0.0%) have expected count less than 5. The minimum expected count is 13.57.

^b^0 cells (0.0%) have expected count less than 5. The minimum expected count is 15.77.

^c^0 cells (0.0%) have expected count less than 5. The minimum expected count is 14.30.

^d^0 cells (0.0%) have expected count less than 5. The minimum expected count is 8.43.

^e^0 cells (0.0%) have expected count less than 5. The minimum expected count is 12.83.

^f^0 cells (0.0%) have expected count less than 5. The minimum expected count is 14.67.

### Sanger sequencing

To perform the Sanger sequencing, the purified RNAs were amplified by PCR. The representative amplicons were then subjected to Sanger sequencing with BigDye™ Terminator v3.1 Cycle Sequencing Kit (Thermo Fisher Scientific) in Applied Biosystems SeqStudio genetic analyzer^28^. The ab1 files from the Sanger sequencing were analyzed using the Sequencing Analysis Software V6.0 (Thermofisher, USA). NCBI BLAST was performed initially and the alignment to SARS-CoV-2 spike gene was also checked in MEGA7 (https://www.megasoftware.net/).

### Data analysis

The data analysis was performed in IBM SPSS version 26.0. A Heatmap was generated with SARS-CoV-2 variants of concern. All Sanger sequence positive samples were considered as gold standard. The Sensitivity, Specificity, Positive Predictive Value (PPV), Negative Predictive Value (NPV) were calculated according to Trevethan 2017^29^ using the following formulae,

- $${\text{Sensitivity }} = \, \left[ {{\text{a}}/\left( {{\text{a}} + {\text{c}}} \right)} \right] \, \times { 1}00$$
- $${\text{Specificity }} = \, \left[ {{\text{d}}/\left( {{\text{b}} + {\text{d}}} \right)} \right] \, \times { 1}00$$
- $${\text{Positive Predictive Value }}\left( {{\text{PPV}}} \right) \, = \, \left[ {{\text{a}}/\left( {{\text{a}} + {\text{b}}} \right)} \right] \, \times { 1}00$$
- ${\text{Negative Predictive Value }}\left( {{\text{NPV}}} \right) \, = \, \left[ {{\text{d}}/\left( {{\text{c}} + {\text{d}}} \right)} \right] \, \times { 1}00$Whereas, a = True positive; b = False positive; c = False negative; d = True negative.

Kappa Agreement, Friedman’s Analysis of Variance (ANOVA) was also performed. The P value reported was 2-sided and considered to be statistically significant at the alpha value < 0.001.

### Ethical approval

All patients’ samples were collected from the left-over nasopharygeal specimens after performing the diagnostic surveillance. All participants or their legal guardians were informed about the study and informed consents were taken over telephone. The study was approved by the ethical review committee (ERC) of Jashore University of Science and Technology, Bangladesh *(Reference: ERC/FBS/JUST/2020-45, Date: 06/10/2020).* We performed all the experiments according to the relevant guidelines and regulations.

## Supplementary Information

Supplementary Information.

## Data Availability

The datasets generated and analyzed during the current study are available in the GISAID repository, [EPI_ISL_12081540, EPI_ISL_12081536, EPI_ISL_12081537, EPI_ISL_12081534, EPI_ISL_12081535, EPI_ISL_12081532, EPI_ISL_12081533, EPI_ISL_12081530, EPI_ISL_12081531, EPI_ISL_12081538, EPI_ISL_12081539, EPI_ISL_12083399, EPI_ISL_12083410, EPI_ISL_12083398, EPI_ISL_12083397, EPI_ISL_12083396, EPI_ISL_12083395, EPI_ISL_12083394, EPI_ISL_12083393, EPI_ISL_12083392, EPI_ISL_12083407, EPI_ISL_12083406, EPI_ISL_12083405, EPI_ISL_12083404, EPI_ISL_12083403, EPI_ISL_12083402, EPI_ISL_12083401, EPI_ISL_12083400, EPI_ISL_12083409, EPI_ISL_12083408, EPI_ISL_12083391, EPI_ISL_12083390, EPI_ISL_11936380, EPI_ISL_12055010, EPI_ISL_12055011, EPI_ISL_12055009, EPI_ISL_12055007, EPI_ISL_12055029, EPI_ISL_12055008, EPI_ISL_12055027, EPI_ISL_12055006, EPI_ISL_12055028, EPI_ISL_12055025, EPI_ISL_12055026, EPI_ISL_12055023, EPI_ISL_12055024, EPI_ISL_12055021, EPI_ISL_12055022, EPI_ISL_12055020, EPI_ISL_12055018, EPI_ISL_12055019, EPI_ISL_12055016, EPI_ISL_12055017, EPI_ISL_12055014, EPI_ISL_12055015, EPI_ISL_12055012, EPI_ISL_12055013].
