# Supplementary material for: Evaluation of RT-PCR assays for detection of SARS-CoV-2 variants of concern
Source: Sci Rep. 2023 Feb 9;13:2342. doi: 10.1038/s41598-023-28275-y (PMC9910272; doi:10.1038/s41598-023-28275-y)

Supplementary Table 1: Thermal cycling conditions and detection targets for different RT-PCR Kits. RT-PCR; Real-Time Polymerase Chain Reaction.

| PCR Kit Name | Holding Stage | Reverse Transcription Stage | RT Inactivation/ Initial denaturation stage | Annealing and Extension (Data Collection) | | Holding Stage | Cycles for amplification | Detection of Channels and target genes |
|---|---|---|---|---|---|---|---|---|
| Maccura (SARS-CoV-2 Fluorescent PCR Kit) | - | 55°C for 15 minutes | 95°C for 2 minutes | 95°C for 15 seconds | 58°C for 35 seconds | 40°C for 10 seconds | 40 cycles | FAM(ORF), ROX(E), Cy5(N), HEX/VIC(IC) |
| A*STAR FORTITUDE KIT 2.0 | - | 48°C for 15 minutes | 95°C for 2 minutes | 95°C for 15 seconds | 59°C for 35 seconds. | - | 42 cycles | FAM (NSP-1) HEX (IC) |
| Da An Gene Co. Ltd. Of SunYat-Sen University | - | 48°C for 15 minutes | 95°C for 15 minutes | 94°C for 15 seconds | 55°C for 45 seconds | - | 45 cycles | FAM(N), VIC (ORF1ab), Cy5 (IC) |
| Novel Coronavirus (2019-nCoV) Nucleic Acid Diagnostic Test (PCR-Fluorescent Probing) from Sansure Biotech | | 50°C for 30 minutes | 95°C for 1 minutes | 95°C for 15 seconds | 60°C for 30 seconds | 25°C for 10 seconds | 42 cycles | FAM (ORF), ROX(N), Cy5(IC) |

| | | | | | | | | |
|---|---|---|---|---|---|---|---|---|
| SYBR-Green method developed in our Laboratory | - | 58°C for 12 minutes | 95°C for 3 minutes | 95°C for 15 seconds | 62°C for 25 seconds | Melting curve stage from 60°C to 95°C. | 44 cycles | SYBR (N, E, Beta Actin as internal control), Detected by Melting Curve Analysis. |
| TaqPath™ COVID-19 CE-IVD RT-PCR Kit | 25°C for 2 minutes | 53°C for 10 minutes | 95°C for 2 minutes | 95°C for 3 seconds | 60°C for 30 seconds | - | 40 cycles | FAM (ORF), ABY(S), VIC(N), JUN (MS2) [Specified for Quantstudio 3 RT-PCR system] |

Supplementary Table 2: Specific standard of the RT-PCR diagnostic kits for result interpretation; RT-PCR: Real-Time Polymerase Chain Reaction.

| Kit name | Positive | Negative | Inconclusive |
|---|---|---|---|
| Maccura (SARS CoV-2 fluorescent PCR kit) | Cycling Threshold (Ct Value) ≤ 38 and obvious amplification curve in FAM and any of ROX, Cy5, HEX/VIC Channel is considered positive. | Cycling Threshold (Ct Value) >38 and no amplification curve in FAM and any of ROX, Cy5 but an obvious amplification curve in HEX/VIC Channel is considered negative. | Cycling Threshold (Ct Value) undetected and no amplification curve in any of FAM, ROX, Cy5, HEX/VIC Channel is considered invalid or retest for the sample. |
| A*Star Fortitude kit 2.0 | Cycling Threshold (Ct Value) < 40 and obvious amplification curve in FAM and Cycling Threshold (Ct Value) < 40 or undetected in HEX channel is considered as positive. | Cycling Threshold (Ct Value) undetermined, no amplification curve in FAM and Cycling Threshold (Ct Value) < 40 in HEX channel is considered as negative. | Cycling Threshold (Ct Value) undetermined in both FAM or HEX channels is regarded as invalid result and needs to re-test. |
| Da An Gene Co. Ltd. of SunYat-Sen University | If the sample has obvious amplification curve in the FAM and VIC channels and Ct value ≤ 40, it can be judged that the sample is considered positive. | If the test sample has no amplification curve in the FAM and VIC channels, there is an amplification curve in the Cy5 channel, then the sample can be considered as negative. | When the internal standard result is negative, if the FAM and VIC detection channels of the test tube are also negative, then the test is considered invalid. |
| Novel Coronavirus (2019-nCoV) Nucleic Acid Diagnostic Test (PCR-Fluorescent Probing) from Sansure Biotech. | Cycling Threshold (Ct Value) ≤ 40 of any of two channels FAM or ROX is considered positive. | Cycling Threshold (Ct Value) > 40 or undetermined for any of the two channels FAM and ROX and obvious amplification curve for Cy5 is considered negative. | If there is no obvious amplification curve in any of the three target genes is considered invalid. |

| | | | |
|---|---|---|---|
| SYBR-Green Based detection primers designed in our Laboratory | Obvious peaks in the derivative plot at 82°C ± 1 for N Gene, at 78°C ±1 for E gene and 86°C ±2 for housekeeping gene (Beta-actin) is considered positive. | No peak or a single peak 10,000 in the derivative plot for N Gene, E gene but obvious peak at 86°C ±1 for housekeeping gene (Beta-actin) is considered negative. | No peak in the derivative plot for N Gene, E gene or for housekeeping gene (Beta-actin) is considered invalid and needs to be retested. |
| TaqPath™ COVID-19 CE-IVD RT-PCR Kit | Amplification curve for two or more SARS-CoV-2 targets (FAM, ABY, VIC) irrespective of JUN channel is considered as positive. | No amplification curve for any of the SARS-CoV-2 targets (FAM, ABY, VIC) but of JUN channel is considered as negative. | No amplification curve for any or amplification curve for one SARS-CoV-2 target (FAM, ABY, VIC) irrespective of JUN channel is considered as invalid or inconclusive. |

Supplementary Figure: Analysis of the results of SYBR-Green Method.

(a) Figure of the amplification plot shown by the samples tested.

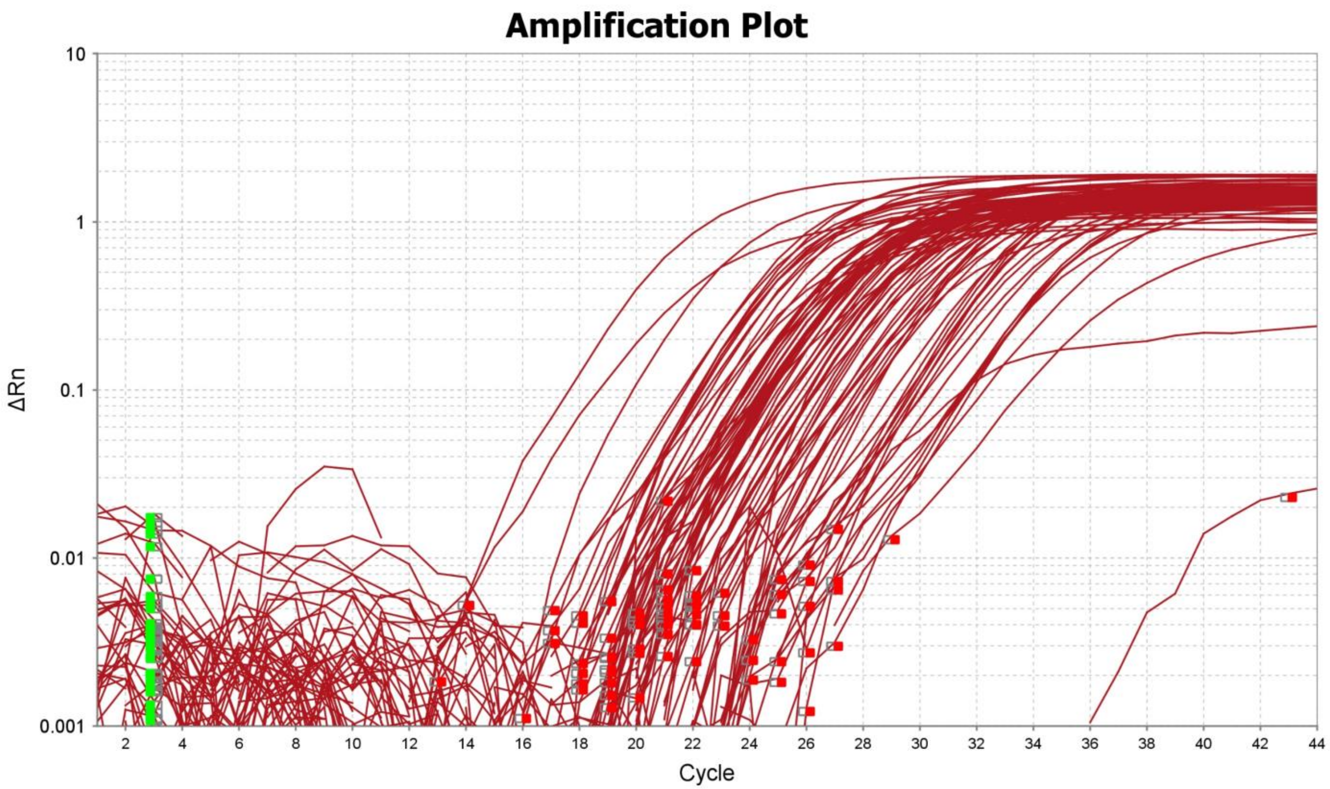

(b) Figure of the melting curve plot shown by the positive samples for different target genes.

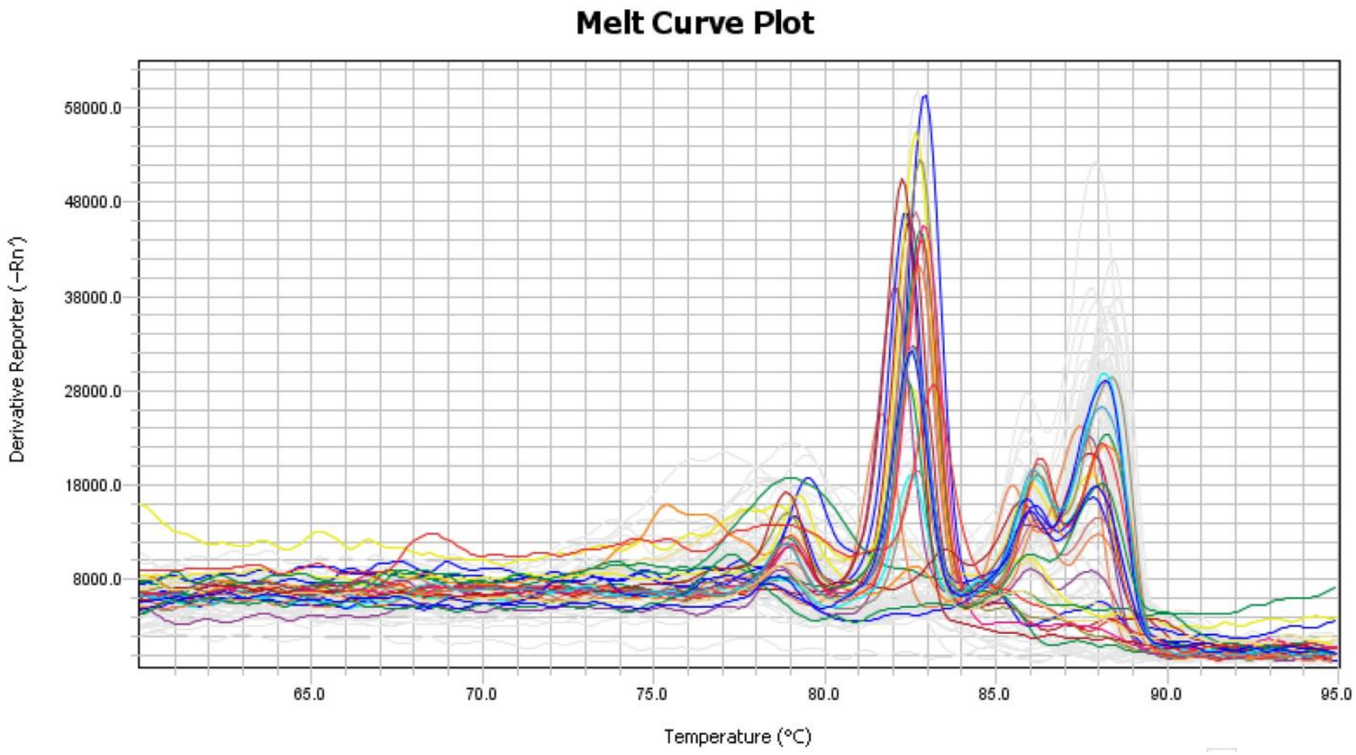

(c) Figure of the melting curve plot shown by the negative samples for different target genes.

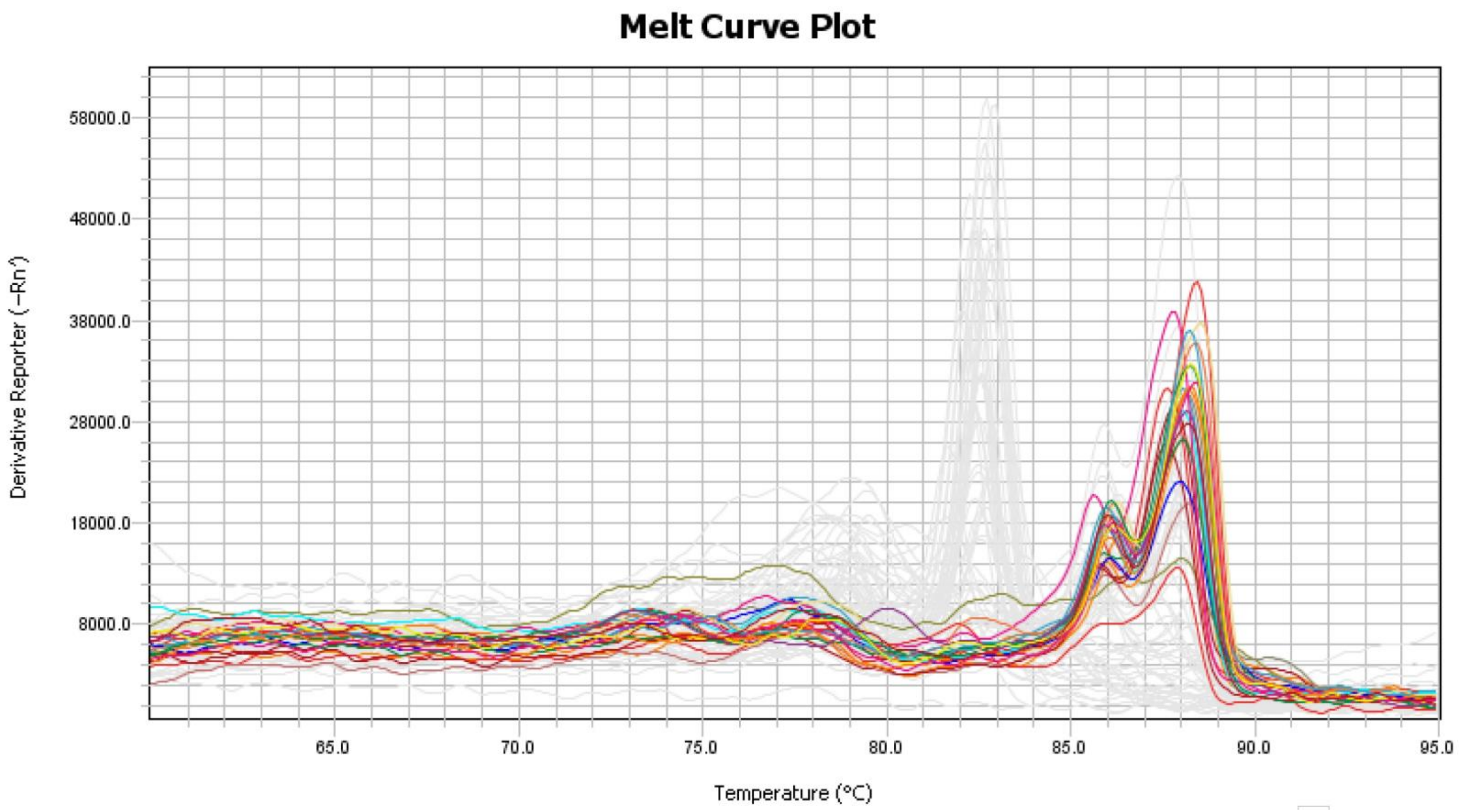

Supplement: Supplementary file 1 — Supplementary Information. [file 41598_2023_28275_MOESM1_ESM.pdf]
